# Optical gearbox enabled versatile multiscale high-throughput multiphoton functional imaging

**DOI:** 10.1038/s41467-022-34472-6

**Published:** 2022-11-02

**Authors:** Jianian Lin, Zongyue Cheng, Guang Yang, Meng Cui

**Affiliations:** 1 School of Electrical and Computer Engineering, Purdue University, West Lafayette, IN 47907 USA; 2 Bindley Bioscience Center, Purdue University, West Lafayette, IN 47907 USA; 3 Department of Anesthesiology, Columbia University Irving Medical Center, New York, NY 10032 USA; 4 Department of Biology, Purdue University, West Lafayette, IN 47907 USA

**Keywords:** Fluorescence imaging, Optical imaging, Ca2+ imaging, Multiphoton microscopy

## Abstract

To understand the function and mechanism of biological systems, it is crucial to observe the cellular dynamics at high spatiotemporal resolutions within live animals. The recent advances in genetically encoded function indicators have significantly improved the response rate to a near millisecond time scale. However, the widely employed in vivo imaging systems often lack the temporal solution to capture the fast biological dynamics. To broadly enable the capability of high-speed in vivo deep-tissue imaging, we developed an optical gearbox. As an add-on module, the optical gearbox can convert the common multiphoton imaging systems for versatile multiscale high-throughput imaging applications. In this work, we demonstrate in vivo 2D and 3D function imaging in mammalian brains at frame rates ranging from 50 to 1000 Hz. The optical gearbox’s versatility and compatibility with the widely employed imaging components will be highly valuable to a variety of deep tissue imaging applications.

## Abstract (web summary)

The authors develop an optical gearbox to accelerate the laser scanning microscopes, achieving flexible adjustment of imaging frame rate from tens of Hz to 1 kHz. The technology is validated through in vivo functional imaging of mice brains.

## Introduction

To reveal the mechanism of complex biological systems, we need to observe the cellular dynamics at sufficient spatiotemporal resolutions. Fast biological events such as neuronal activity often demand millisecond scale temporal resolution^1–3^. Thanks to the recent advance in genetic function indicators^4–9^, in vivo function imaging at a near kilo Hertz frame rate, has become possible, which demands high-throughput imaging methods. Although the parallel recording via cameras can meet such frame rate requirements^10–19^, the light scattering induced noise crosstalk and the exponential decay of the ballistic signal component severely limit their practical imaging depth. For deep tissue recording in live mammalian models, multiphoton microscopy remains the gold standard^20^. However, the common multiphoton recording systems widely used in the research community are often too slow to keep up with these fast dynamics, which has become a major obstacle to broadly enabling high-speed recording in various research applications. Recently, several new techniques have been demonstrated to achieve high frame rate recording^21–37^. Despite the great advances in the measurement throughput, there are often various tradeoffs in the performance of multiphoton imaging, such as imaging depth, contrast, noise crosstalk, spatial resolutions, and motion artifact. From the perspective of technology dissemination, a major challenge above all is that the implementation of these novel solutions often involves highly specialized niche components, which are incompatible with the widely employed imaging systems. Together, these factors limit the implementation of these novel solutions to only a few labs.

The in vivo imaging speed of multiphoton systems depend on the excitation power, pulse repetition rate, scanner speed, excitation numerical aperture (NA), fluorescence signal collection efficiency, fluorophore concentration within the focal volume, fluorophore brightness and bleaching rate, photodamage threshold, and thermal limit^38^. With the choice of the high-performance fluorescence function indicators and the optimized excitation condition and detection efficiency, the choice of laser repetition rate and the achievable scanner speed has a major impact on the imaging rate. The widely employed Ti:Sapphire oscillator operates near 80 MHz, which provides an acceptable balance between the nonlinear excitation efficiency and the maximum sampling rate. With the limited power allowed in live animal studies, a higher repetition rate in principle allows a greater maximum sampling rate albeit at the cost of reduced nonlinear excitation efficiency and increased chances of emission signal crosstalk due to the nanosecond scale fluorescence lifetime. In fact, if we can utilize the 80 MHz sampling efficiently, it may yield up to 283 × 283 pixels at 1000 Hz and 632 × 632 pixels at 200 Hz, acceptable for many applications. Fortunately, this common 80 MHz repetition rate matches quite well with the commonly used fast laser scanners such as resonant galvo and polygon scanners. Take the common 5 mm 8 kHz resonant galvo as an example, the average speed can cover ~40 million spatially resolvable spots per second, which at the Nyquist sampling limit matches the 80 MHz sampling rate. For the case of polygon scanners^39^, its throughput can more than double that of the resonant galvo with the additional benefit of purely linear scanning speed.

A key problem of the common fast scanners is that they achieve their maximum throughput (i.e. diffraction-limited spots per second) only for large-angle scanning. For resonant galvo scanners, the mirror acceleration is linearly proportional to the scan range and quadratically proportional to the scan frequency. However, the scanning throughput is proportional to the product of the scan range and the scan frequency. Therefore, with the peak acceleration limited, increasing the line rate will inevitably lead to reduced overall throughput. For example, the higher rate 12 kHz resonant galvo scanner can only achieve half of the scan range of that of the 8 kHz version, resulting in reduced data throughput. For polygon scanners, given the same rotor spinning speed, increasing the mirror facet number can lead to a faster line frequency, which comes at the cost of increased beam divergence (l/D, where l is the wavelength and D is the beam diameter) and reduced throughput due to the smaller beam size supported by each mirror facet. Overall, the high scanning throughput of the common laser scanners is only achievable for large-angle scanning. For high frame rate imaging, the obtainable image therefore becomes a long strip with few lines^40^, impractical for most applications.

To adapt the commonly employed laser sources and scanners for high frame rate imaging, we have developed an optical gearbox to overcome the scan frequency and scan throughput tradeoff of common scanners. The core idea is to let the common optical scanners operate at the optimal condition that yields the highest throughput and to leverage the optical gearbox to tailor the scanning pattern to a usable form for high frame rate applications. The basic function of the optical gearbox is to enable the flexibility to convert a large-angle scanning into a user-defined number of short-range scanning. In such a way, zooming in by a factor of n leads to an n times faster line rate and an n^2^ times faster frame rate, which is n times faster than that of the conventional multiphoton imaging systems. The essence of an optical gearbox contains three components: optical path separation, path spacing amplification, and sequential input to the scanner. Take a 24 optical degree scanner as an example, we first employ a fast-switching device (e.g. acousto-optic deflector, AOD) to switch the laser beam towards 8 different angles (i.e. achieving optical path separation). Due to the limited time-bandwidth product of the AOD, the angular spacing (number of angularly resolvable spots) between the adjacent paths is very limited. In the second step, we employ optical components to amplify the angular spacing between these 8 paths before the beams arrive on the laser scanner such that their angular spacing on the scanner is equal to 3 optical degrees (i.e. achieving path spacing amplification). In the final step, we synchronize the sequential beam path switching with the scanning of the scanner such that a single full-range line scan becomes 8 sequential line scans, each with 1/8 of the original scan range. Thus, we obtain 8 times line rate acceleration without a tradeoff in throughput. As the path switching is electronically controlled, we have the freedom to adjust the line rate or scan range at will. For example, instead of visiting all 8 paths sequentially, we can command the laser beam to visit the odd number of paths and therefore accelerate the line rate by a factor of 4, each with 1/4 of the full scan range. With the throughput conservation and the rate and range flexibility considered, this mechanism bears similarity to that of the mechanical gearbox. Thus, we named this method optical gearbox. In this work, we employ the optical gearbox-based imaging system for 2D and 3D multiscale measurement of fast biological events within live animals.

## Results

### System design for optical gearbox based two-photon imaging

**Fig. 1:**
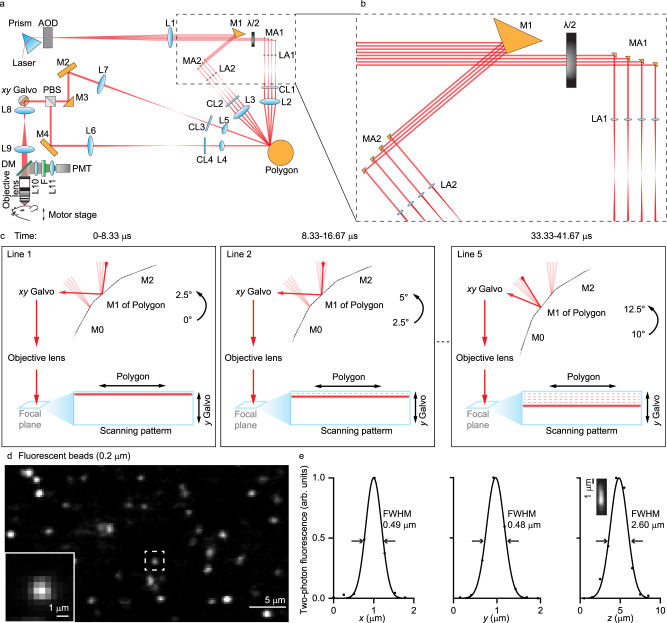
System design of the polygon-based optical gearbox. **a** The optical design of the optical gearbox based two-photon fluorescence imaging system. AOD acousto-optic deflector; L1-11, optical lenses; λ/2, half-wave plate; MA mirror array, LA lens array, CL cylindrical lenses, M1-4 mirrors, PBS polarizing beam splitter, DM dichroic beam splitter, F fluorescence bandpass filter, PMT photomultiplier tube. **b** The zoomed-in view of the mirror and lens array. **c** The design of the dual-path configuration for the polygon scanner. **d** Two-photon images of 0.2 µm fluorescence beads in agar. **e** Cross-sectional plots of the focal profile. FWHM full width at half maximum.

To achieve highly uniform scanning, we implemented the optical gearbox system (Fig. 1a, Supplementary Movie 1) using a polygon scanner (SA34, Cambridge Technology). For the optical path separation, we employed an AOD (DTSXY400, AA Opto-Electronic) to switch the laser beam between eight paths with a microsecond transition time. As the AOD-generated angular scanning range was small, the spatial chirp of the AOD can be uniformly compensated by a single Brewster optical prism made of H-ZF72A glass with a 56-degree apex angle (Supplementary Fig. 1). The path spacing amplification involved two stages. In the first stage, the closely spaced optical beam paths were reflected by the mirror arrays positioned near the focal plane of L1 (Fig. 1b). The mirror spacing was small (2.1 mm) in the direction perpendicular to the incident beam and was large (9 mm) in the direction along the incident beam. With the low NA (~0.001) of L1, the optical path length variation introduced by the mirror array was much less than the tens of centimeters Rayleigh length of the laser beam. After this stage of spacing amplification, the spacing was large enough to allow the position of the lens array, which focused the laser beam into small spots and greatly increased the path spacing. We designed a compact assembly, which provided angular and spatial tuning for the mirror array and lens array, respectively, and could lock the positions of the mirrors and lenses with long-term stability (Supplementary Fig. 2). Through telecentric relay lenses, these foci became the array of beam paths sequentially incident on the polygon scanner with uniformly spaced incident angles. We also positioned cylindrical lenses before (CL1, 2) and after (CL3, 4) the beam arrived at the polygon scanner for optical wobble correction^41^.

Due to the traveling nature of the polygon mirror facet, the commonly achievable duty cycle is often far from unity. To overcome this limitation and achieve ~100% duty cycle, we developed a dual-path configuration. Specifically, we used a knife-edge mirror to separate the beam paths into two halves (Fig. 1b). Temporally, the first half sequentially arrived near the center of the polygon mirror. Before the mirror boundary could move across the incident beam, the laser was switched to the second half, which again was at regions far away from the mirror boundaries (Fig. 1c). The interleaved sequential operation tightly synchronized with the polygon scanning achieved near 100% duty cycle. The two halves of the beam paths were combined by a polarizing beam splitter (PBS) and then relay imaged to a galvo scanner (8315 K, Cambridge Technology), which was imaged to the back focal plane of the imaging objective lens. The PBS-based beam combination was of negligible power loss and well compatible with two-photon fluorescence imaging. For polarization-sensitive imaging tasks, non-polarizing beam splitters could be used, which albeit could only use half of the laser power. The excited fluorescence signal was directed by high-etendue lenses onto a photomultiplier tube (PMT), whose signal was sampled at 80 MHz. The data acquisition was precisely synchronized to the polygon scanner with optical feedback (Supplementary Fig. 3a). We developed a user-friendly graphic user interface (GUI) for system control and image acquisition (Supplementary Fig. 3b), whose functionality resembled that of the commonly used open-source multiphoton acquisition software *ScanImage*. Using 0.2 µm fluorescence beads, we quantified the two-photon spatial resolution (Fig. 1d). Numerical curve fitting showed 0.48, 0.49 and 2.6 µm in the transverse and axial resolutions (Fig. 1e), respectively.

### Optical gearbox enabled high-speed 2D imaging

**Fig. 2:**
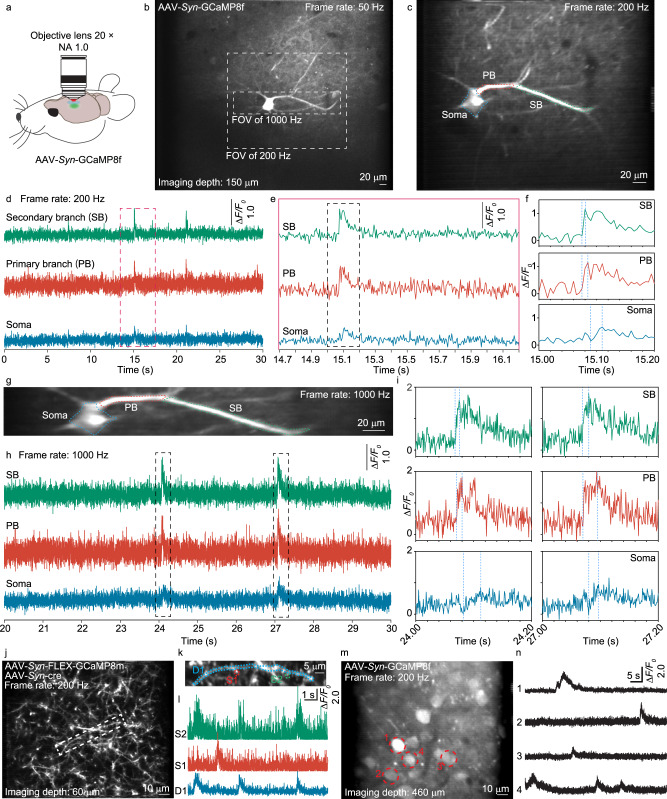
In vivo 2D high-speed imaging of neuronal structures. **a** Experiment configuration. The high-speed imaging was repeated independently in 13 mice with similar results. NA numerical aperture. **b** Large field of view imaging with 4x gearbox setting, representative of 4 FOV from 2 mice. FOV, field of view. **c** Imaging with 8x gearbox setting and 200 Hz frame rate, representative of 24 FOV from 10 mice. PB primary branch, SB secondary branch. **d** Calcium transients from neuronal structures at 200 Hz. **e** Zoomed-in view of the red dashed box in (**d**). **f** Zoomed-in view of the black dashed box in (**e**). **g** Imaging with 8x gearbox setting and 1000 Hz frame rate, representative of 7 FOV from 6 mice. **h** Calcium transients from neuronal structures at 1000 Hz. **i** Zoomed-in view of the dashed box in (**h**). **j** Imaging fine dendritic structures at 200 Hz, representative of 17 FOV from 6 mice. **k** Zoomed-in view of the dashed box in (**j**). D dendrite, S dendritic spine. **l** Calcium transients from dendrite and dendritic spines. **m** Deep tissue imaging of layer V neurons at 460 µm depth, representative of 4 FOV from 3 mice. **n** The calcium transients from the labeled soma in (**m**).

To evaluate the 2D imaging performance of the gearbox system, we imaged the brain of live mice that expressed the recently developed fast calcium indicator GCaMP8f^9^ (Fig. 2, Supplementary Movie 2). With the polygon scanner providing 15 kHz intrinsic line rate, we obtained 120 kHz and 60 kHz line rates with the gearbox configured for 8x (visit all 8 paths) and 4x (visit path 1, 3, 5, 7 only) acceleration. We first used the 4x configuration to obtain large-field images (Fig. 2b) and then zoomed in with the 8x configuration for faster measurement at 200 Hz (Fig. 2c). The high frame rate allowed resolving the timing difference between the dendritic branches and somata (Fig. 2d–f), which showed that the calcium spike propagated from the dendritic branch to the soma. The GCaMP8f features a short rise time. On the dendrites, the rising time appeared to challenge the 5-millisecond sampling interval. To better sample the rising transient, we shortened the frame length and further increased the frame rate to 1 kHz (Fig. 2g–i). Based on the raw data recorded at 1 kHz sampling rate, we evaluated the effects of reduced sampling rates (Supplementary Fig. 4). On somata, calcium transients could be captured with 50 Hz sampling rate. On dendrites, 200 Hz or higher rate was required to sample temporally adjacent spikes. As the gearbox has no tradeoff in imaging resolution and contrast, it also enabled the recording of fine dendritic spines (Fig. 2j–l). For larger structures such as somata, the gearbox system could enable high-speed recording at a large imaging depth (layer V of mouse cortex, Fig. 2m, n).

### Optical gearbox enabled 3D imaging

**Fig. 3:**
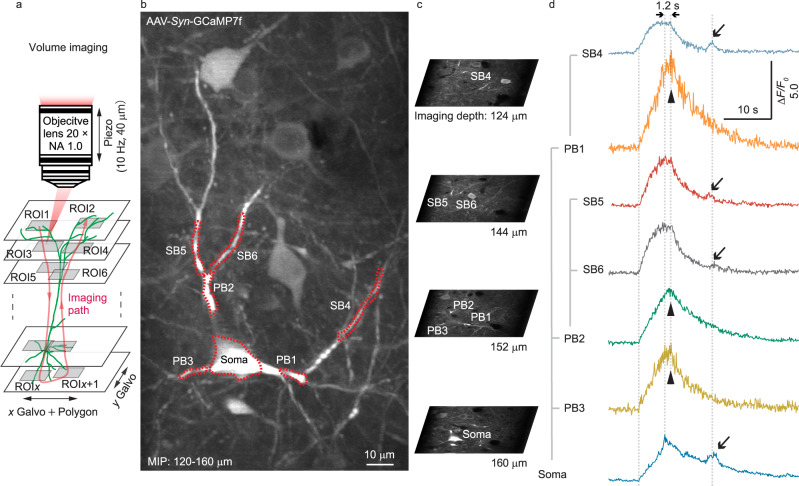
In vivo 3D calcium imaging of neuronal structures. **a** Experimental configuration of the 3D calcium recording. NA numerical aperture, ROI region of interest. **b** Maximum intensity projection of the imaging volume along the axial direction. PB primary branch, SB secondary branch, MIP maximum intensity projection. The experiment was repeated independently in 4 mice with similar results. **c** 2D images at various depths. **d** Calcium transients from the various regions of the neuronal structures.

Spreading the 2D image frames over a 3D volume, we enabled the recording of signal propagation on 3D neuronal structures. Experimentally, we combined the axial movement of the piezo driven objective lens with the transverse positioning of the 2D image frame by the 2-axis galvo scanner to cover 20 regions of interest (2D image slices) in 3D at a 10 Hz volume rate (Fig. 3, Supplementary Movie 3). Such a configuration provided the flexibility to study 3D extended structures. As an example, we performed calcium imaging on the soma and dendrites of GCaMP7f expressing neurons in the brain of awake mice. First, we employed the 4x gearbox setting and the 2-axis galvo scanner to acquire large-volume 3D structure images (Fig. 3b). Next, we employed a customized software with GUI to select the regions of interest in 3D (Fig. 3c). Based on the user selection, the software configured the piezo scanning path and the 2-axis galvo scanner driving voltages and started the time-lapse volumetric recording. From the 3D images, we extracted the calcium transients from the soma, primary and secondary dendritic branches, which revealed distinct temporal dynamics. In certain cases, the calcium transients in secondary branches preceded the somatic signals, and the calcium decay was prolonged in secondary branches, likely due to the backpropagating action potential^42^ (Fig. 3d, triangular arrowheads). In another case, the calcium transient could be observed simultaneously from the soma and the secondary dendritic branches, but not from the primary branches, likely due to the negative regulation^43^ in the primary branch (Fig. 3d, arrows). Such observations highlighted the importance of 3D volumetric recording and demonstrated the versatility of the gearbox system.

### Optical gearbox enabled blood flow imaging

**Fig. 4:**
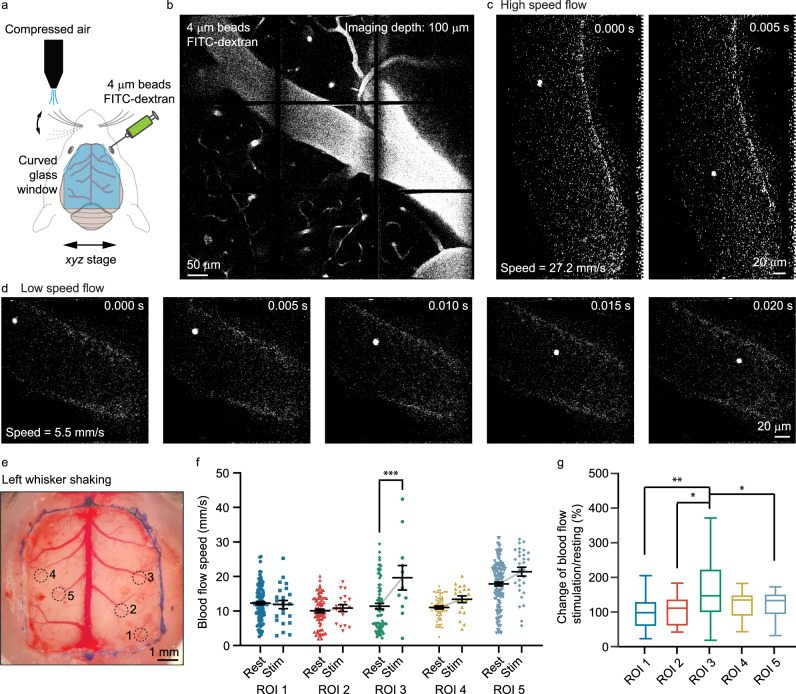
High-speed large-scale functional blood flow imaging. **a** Experimental configuration with the whisker stimulation and fluorophore injection. FITC fluorescein isothiocyanate. **b** Large field imaging of the blood vessel network. **c** Imaging fluorescence beads in fast blood flow with a 200 Hz frame rate. **d** Imaging fluorescence beads in slow blood flow with a 200 Hz frame rate. **e** Top view of the large area cranial window and the regions of interest. **f** Blood flow speed before and during the whisker stimulation across the five regions of interest. Data are represented as mean ± standard error of the mean. ***P = 0.0007. Stim stimulation, ROI region of interest. **g** Statistical analysis of the blood flow speed variation. For boxplots, minima and maxima are shown as the bounds of whiskers, and the centile, upper and lower quartiles are shown as the middle, top, and bottom lines of the box. **P (ROI 1 vs 3) = 0.0011, *P (ROI 2 vs 3) = 0.0135, *P (ROI 3 vs 5) = 0.04. One-way ANOVA Tukey’s multiple comparisons test in (**f** and **g**). *n* = 108/23, 73/18, 75/11, 61/19, 138/29 (rest/stimulation) speed measurements in ROI 1-5 from one animal, respectively. The experiment was repeated independently in 8 mice with similar results.

Vasculature dynamics play a major role in the function of biological systems^10,44–46^. Conventional multiphoton imaging systems lack the speed to track rapid blood flow. Leveraging the high frame rate of gearbox-based imaging, we could quantify the blood flow with flow speed up to 100 mm/sec sequentially over centimeter-scale brain regions (Fig. 4, Supplementary Movie 4). With the curved glass cranial window^47^, we obtained centimeter-scale optical access to the mouse brain. Using fluorescein isothiocyanate (FITC)-dextran (80 μl, retro-orbital injection), we labeled the blood vessel. To optically track the blood plasma flow, we injected 4 µm fluorescence beads (20 μl, retro-orbital injection), whose Stokes number was ~0.01, much less than unity^48^, thus allowing the beads to closely follow blood flow. As the blood flow rate varied slowly in time, we used a continuous measurement time of two seconds at each location. With the 8x gearbox setting, we imaged the blood flow at a 200 Hz frame rate. From each set of 400 image frames recorded over two seconds, we extracted the blood flow speed based on the bead location variations (Fig. 4b–d). To demonstrate the large-scale functional recording, we used compressed air to stimulate the left whiskers of awake mice and employed the gearbox system to sequentially measure five brain regions repeatedly over time before and during the whisker stimulation (Fig. 4a, e). Statistics showed that the region near the right barrel cortex (ROI 3 in Fig. 4e) showed a significant blood flow rate increase, as expected for the left whisker stimulation (Fig. 4f, g). This study demonstrated the large-scale high-speed blood flow imaging capability of the gearbox system, which may be combined with the calcium imaging to provide a complementary picture of brain activity.

### Extended fluorescence decay time via high-speed scanning

One inherent advantage of such high-rate spatial scanning is that the laser pulses were separated in time and space (spatial spacing of the pulses equal to half of the spatial resolution), allowing dark-state relaxation of the fluorophores, and reducing the photobleaching rate^49^. As an example, we performed in vivo imaging of the dendritic structures of *Thy1*-eGFP mice and compared the photobleaching rate obtained by the gearbox system and by the galvo scanning with the same excitation parameters (Supplementary Fig. 5). Statistical analysis showed a severalfold increase in the fluorescence decay time, which demonstrated the benefit of the gearbox-based high-rate imaging for long-duration recording applications.

## Discussion

In this work, we demonstrated a polygon scanner-based gearbox system. The pixel rate was limited to 80 MHz by the laser source. With the dual-path configuration, the polygon scanner can support up to ~180 MHz pixel rate at the Nyquist sampling limit (Supplementary Discussion 1) with a near 100% duty cycle at 920 nm wavelength. A common trade-off of high-rate scanning is that the dwell time is proportionally reduced. The effective imaging throughput was ultimately determined by the sample brightness. Nonetheless, with the extended fluorescence decay time enabled by the high-rate scanning considered, even for low-brightness samples, it is still preferable to employ high-rate scanning and frame averaging.

A key advantage of the gearbox system is its compatibility with the commonly available hardware components. The employed 80 MHz laser source, polygon, and galvo scanners, 80 MHz digitizers have been widely employed in common high-rate laser scanning microscope systems^50^. In the earlier version of the gearbox system, we also employed a resonant galvo as the main high-speed scanner (Supplementary Fig. 6) although its linearity was inferior to that of the polygon. In addition to the compatibility, the gearbox system offers excellent flexibility. One has the freedom to freely adjust the imaging FOV without losing imaging throughput or duty cycle. In the past, polygon scanners were unfavored for microscopy applications due to their inflexibility despite their great advantages in linearity and throughput. With the help of a gearbox, one can benefit from these advantages without any sacrifice in flexibility. Furthermore, the dual-path configuration we developed overcame the traveling mirror boundary problem of the polygon scanner and enabled a near 100% imaging duty cycle.

Compared to some of the earlier techniques, a key aspect of the gearbox-based high-speed scanning is that the speed gain comes without any loss or spatial variation in focus quality, spatial resolution, and imaging contrast. As a result, the gearbox system can perform functional recording for both large (e.g. somata) and fine structures (e.g. dendrites and spines) deep in the animal brain, which demands a 200 Hz or higher frame rate. The accompanied benefit of such high-rate imaging was that motion correction could be achieved through only rigid image translation, which was far more efficient and robust in comparison to the nonrigid registration required for the low-rate imaging due to its nonnegligible motion within each image frame.

In summary, we developed an optical gearbox to achieve high-rate imaging based on the commonly available imaging hardware. As an add-on module to a laser scanning microscope system, the gearbox can flexibly adjust the imaging frame rate and FOV with the same high data throughput. Different from conventional multiphoton imaging systems, zooming in the image by a factor of n with the gearbox speeds up the imaging by n^2^ times. Experimentally, we demonstrated in vivo 2D and 3D calcium imaging of neurons and 2D blood flow speed imaging. As the gearbox preserved the inherent two-photon focus quality, confinement, and contrast, the high-rate imaging was applicable to fine dendritic structures. Moreover, the spatiotemporally spaced laser pulses prolong the fluorescence decay time during long recording sessions. With its flexibility and compatibility with widely available imaging components, the optical gearbox holds great potential to broadly enable high-rate functional recording in a wide range of applications.

## Methods

### Animal

The research work complied with all relevant ethical regulations and all procedures involving mice were approved by the Animal Care and Use Committees of Purdue University (Protocol Number: 1506001267). The wild-type (WT) C57BL/6 mice (for virus injection), *Thy1*-eGFP M line were purchased from the Jackson Laboratory (Supplementary Table 1). The viruses (AAV1-*Syn*-jGCaMP8f-WPRE, AAV1-*Syn*-FLEX-jGCaMP8m-WPRE, AAV1-*hSyn*-Cre-WPRE-hGH, AAV1-*Syn*-jGCaMP7f-WPRE, AAV1-*Syn*-GCaMP6s) were purchased from Addgene. Mice were housed in Bindley Bioscience Center Animal Facility at Purdue University. The surgical procedures were performed on one to two months old male and female mice.

### Mirror and lens array assembly and alignment

To hold and adjust the micromirror and lens array, we designed and machined a compact device (Supplementary Fig. 2). First, we bonded the prim mirrors onto the L shape holders using UV epoxy. The bottom of the L shape holders was attached to the mainframe by machine screws and spring washers. To align the horizontal direction of the mirrors, we illuminated the mirror array with a collimated beam of light and observed the reflection using a telecentric lens and a camera. If the reflection of the mirrors were in the same direction, its focus would remain in the same spot on the camera. We manually adjusted the horizontal direction of each L shape holder before tightly locking the machine screw at the bottom. For the vertical adjustment, we employed an array of fine adjustment screws which could vertically tilt each mirror. Again, using the focal position on the camera as the feedback, we adjusted each mirror’s vertical orientation before tightly locking the machine screws. We bonded the microlenses onto individual holders using UV epoxy, which were then attached to the mainframe by machine screws and spring washers. To check the alignment, we again used a camera to image the focal spot of each microlens, which was supposed to be on a horizontal line with identical spacing. For precise alignment, we attached each lens holder onto a 3-axis manual stage using the clearance hole on top of the lens holder and positioned the lens holder with micron-level precision before locking its position using two machine screws.

### Design of data acquisition system

The polygon scanner was driven by a brushless DC motor whose rotation speed was precisely synchronized by a pulse train from a function generator via a phase lock loop. For accurate timing control, we employed optical feedback (single-mode fiber source and photodiode with slit) for sensing the scanning mirror rotation. The detected analog optical feedback signal was converted to digital pulses by a comparator. These digital pulses triggered both the data acquisition (one trigger per facet) and the generation of sub-pulses depending on the user configuration. For example, to achieve an 8x gearbox configuration, one optical feedback pulse triggered the data acquisition to start recording and also triggered a function generator to output 8 evenly spaced sub-pulses. These sub-pulses served as the sampling clock for a voltage analog output card that generated the control voltage signals for the AOD and the *y* galvo. The recorded data were then reshaped into 8 lines. The sampling rate of the data acquisition was set to 80 MHz, which was synchronized with the repetition of the laser pulses. As reported by an earlier study^51^, when the sampling rate of the imaging system was close to the Nyquist sampling limit, the sampling process could introduce a noticeable timing jitter. To eliminate such a jitter, we employed a newly developed digital jitter correction method^51^.

To provide a user-friendly interface, we designed the GUI such that its function and appearance were similar to that of the widely used open-source laser scanning microscopy control software *ScanImage* (Supplementary Fig. 3). The GUI allowed users to conveniently set the gearbox zoom setting (e.g. 2x, 4x, 8x), continuously view the real-time images, and record a user-defined number of images over time.

### Whisker stimulation

In the blood flow measurement, we employed compressed air to stimulate the whiskers of mice. To provide a gentle stimulation, the compressed air was first passed through a compressed air regulator (AR25-B, SMC), then a relay voltage-controlled gas valve, before reaching the whiskers which were 1–2 cm away from the air outlet. The air pressure was regulated to between 15 and 18 kPa above atmospheric pressure. The control period of the measurement (no airflow) was 1 min, and the air stimulation period lasted 20 s.

### Data analysis

All the time-lapse images were registered with the averaged image by using mutual information for motion correction. Relative changes in fluorescence (Δ*F/F*_*0*_) were quantified from regions of interest (ROI). *F*_*0*_ was defined as the minimum 10% of each ROI’s fluorescence signal.

### Statistics

All the data in this study are represented as mean ± standard error of the mean or boxplots. For boxplots, minima and maxima are shown as the bounds of whiskers, and the centile, upper and lower quartiles are shown as the middle, top, and bottom lines of the box. The Shapiro–Wilk normality test was performed for all the data. The one-way ANOVA Tukey’s multiple comparisons test was selected for comparing multiple groups (passed normality test) in Fig. 4f, g. *P* < 0.05 is recognized as statistically significant. All statistical analyses were performed using *GraphPad Prism (8.0.2)*, and the curve in Supplementary Fig. 5b was fitted using *MATLAB (2021a)*. No results of the successful acquisition from images and measurements were excluded and filtered. The experiment did not include randomization and blinding. n, *P* values, and the statistical tests were shown in the figure legends.

### Reporting summary

Further information on research design is available in the Nature Research Reporting Summary linked to this article.

## Supplementary information

Supplementary Information

Description of Additional Supplementary Files

Supplementary Movie 1

Supplementary Movie 2

Supplementary Movie 3

Supplementary Movie 4

Reporting Summary

## Data Availability

The data generated in this study have been deposited in the Science Data Bank database^52^ [10.57760/sciencedb.02730].

## Code availability

The code used in this study is available in the Science Data Bank [10.57760/sciencedb.02764].
