# Supplementary material for: Optical gearbox enabled versatile multiscale high-throughput multiphoton functional imaging
Source: Nat Commun. 2022 Nov 2;13:6564. doi: 10.1038/s41467-022-34472-6 (PMC9630539; doi:10.1038/s41467-022-34472-6)
Supplement: Supplementary file 1 — Supplementary Information [file 41467_2022_34472_MOESM1_ESM.docx]

Supplementary Material for

Optical gearbox enabled versatile multiscale high-throughput
multiphoton functional imaging

Jianian Lin^1, 2^†, Zongyue Cheng^1, 2^†, Guang Yang^3^ and Meng Cui^1, 2, 4*^

^1^School of Electrical and Computer Engineering, Purdue University, West Lafayette, IN 47907, USA

^2^Bindley Bioscience Center, Purdue University, West Lafayette, IN 47907, USA

^3^Department of Anesthesiology, Columbia University Irving Medical Center, New York, NY 10032, USA

^4^Department of Biology, Purdue University, West Lafayette, IN 47907, USA

†These authors contributed equally to this work.

*Correspondence:

Dr. Meng Cui,

(+1) 765-496-1332

Email: [mengcui@purdue.edu](mailto:mengcui@purdue.edu)

This file includes Supplementary Figures 1-6, Supplementary Table 1, and Supplementary Discussion 1.


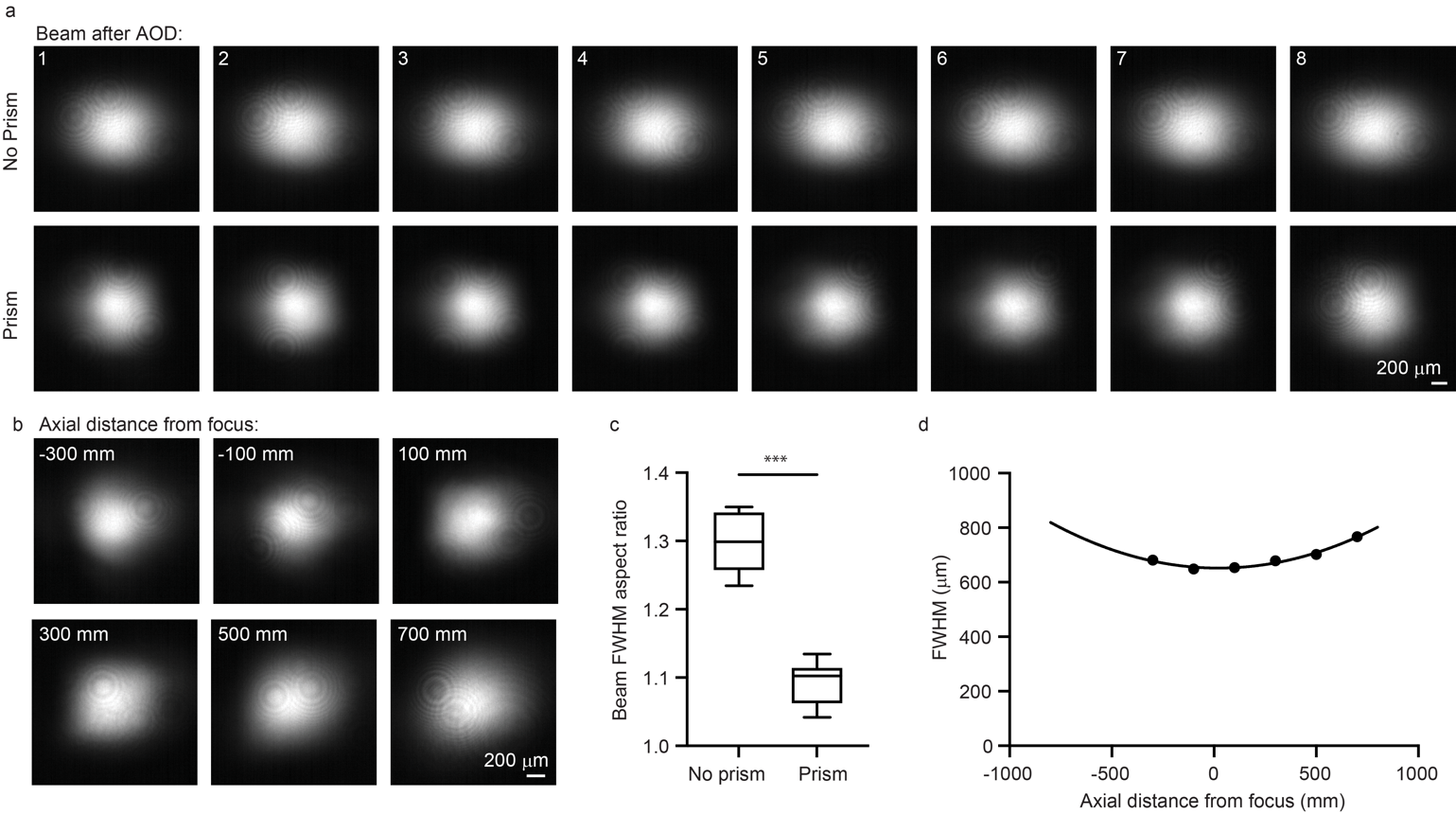


**Supplementary Figure 1 | Optical beam profile after lens L1**. (a) The focus profile at the focal plane of lens L1 measured by a camera for all eight beam paths without and with the prism. AOD, acousto-optic deflector (b) The beam profiles at various distances from the focal plane. (c) The horizontal and vertical aspect ratio quantification for the images in a. ***: *P* = 1.29E-05, Two-tailed paired t-test. n = 8 laser beams. For boxplots, minima and maxima are shown as the bounds of whiskers, and the centile, upper and lower quartiles are shown as the middle, top, and bottom lines of the box. (d) Beam size quantification for the images in b as a function of distance from the focus.


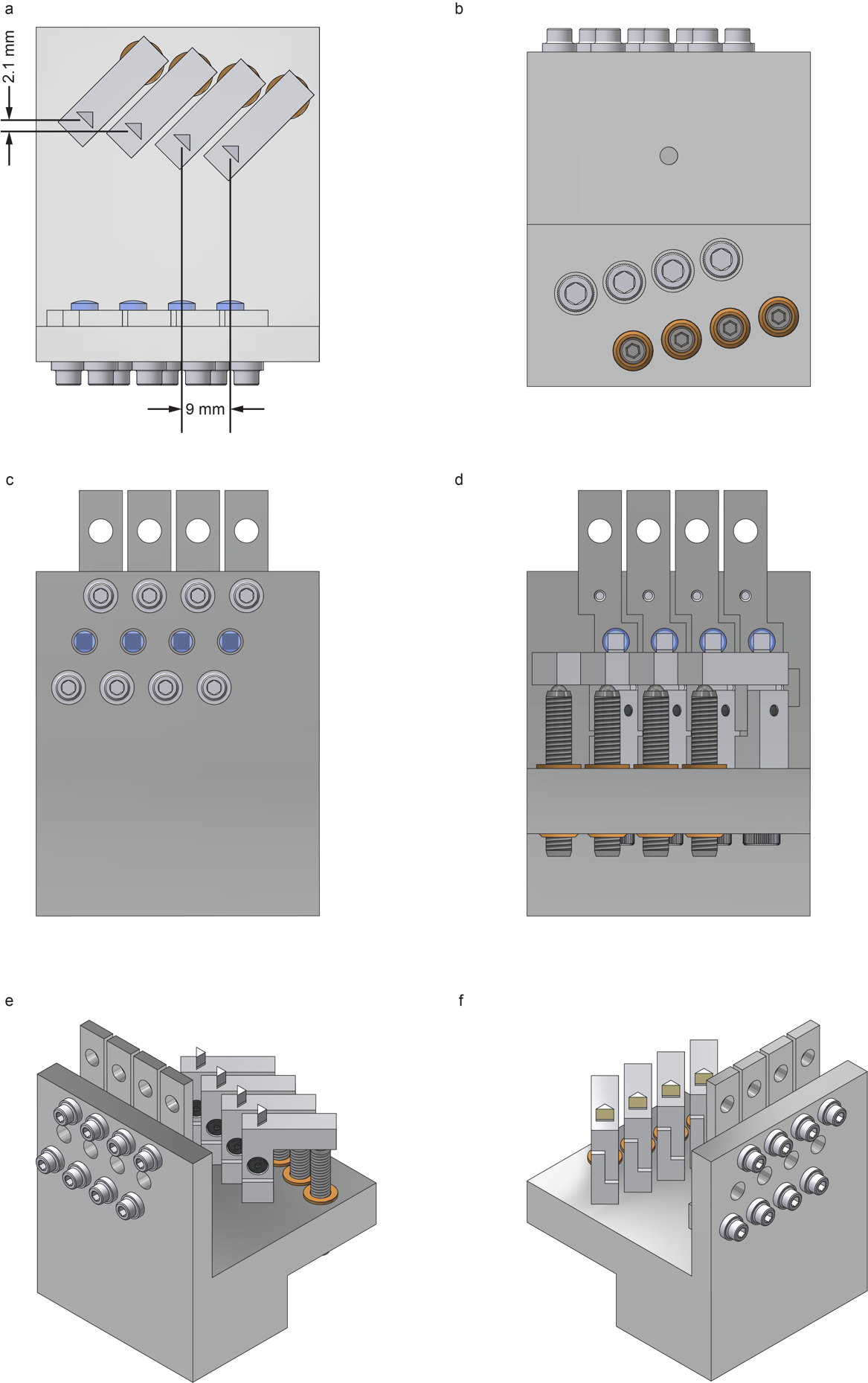

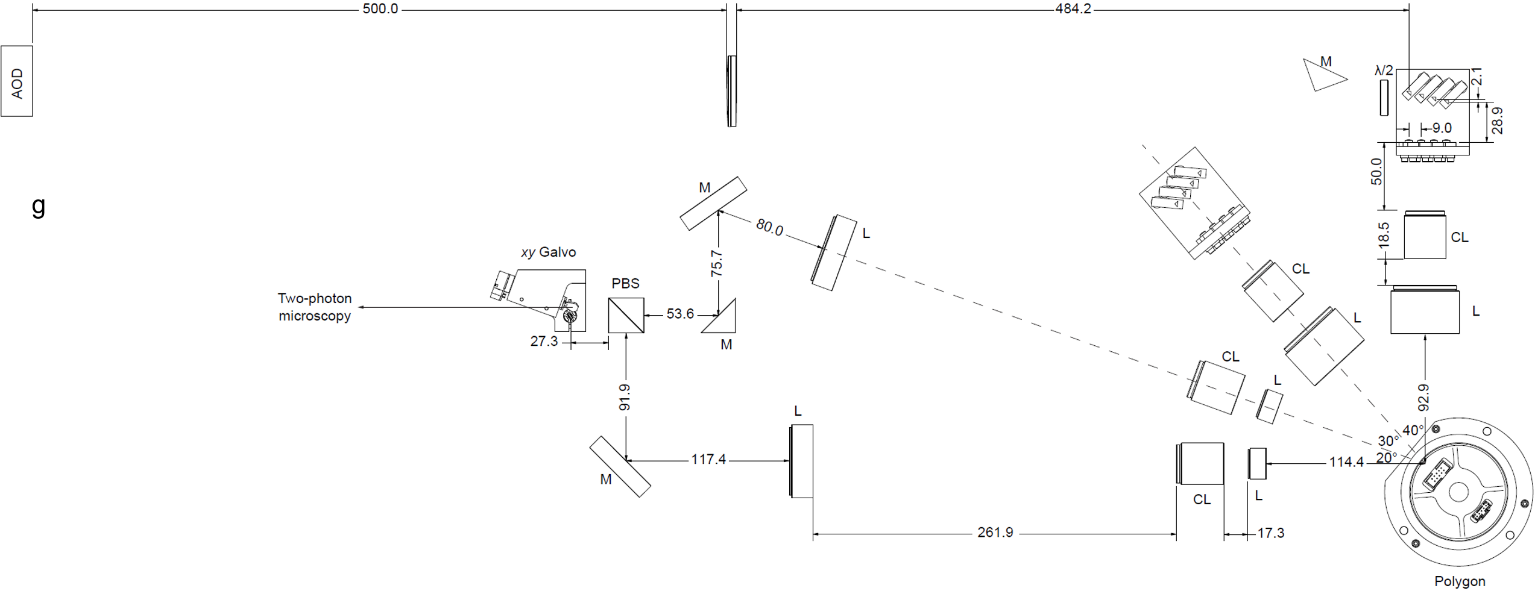


**Supplementary Figure 2 | Mechanical design of the mirror array and lens array holder**. (a-f) Top, bottom, front, back, right front, left front view of the holder, respectively. (g) The dimension of the array holder with respect to the overall gearbox system. Dimension unit, mm. AOD, acousto-optic deflector; L, optical lenses; λ/2, half-wave plate; CL, cylindrical lenses; M, mirrors; PBS, polarizing beam splitter.


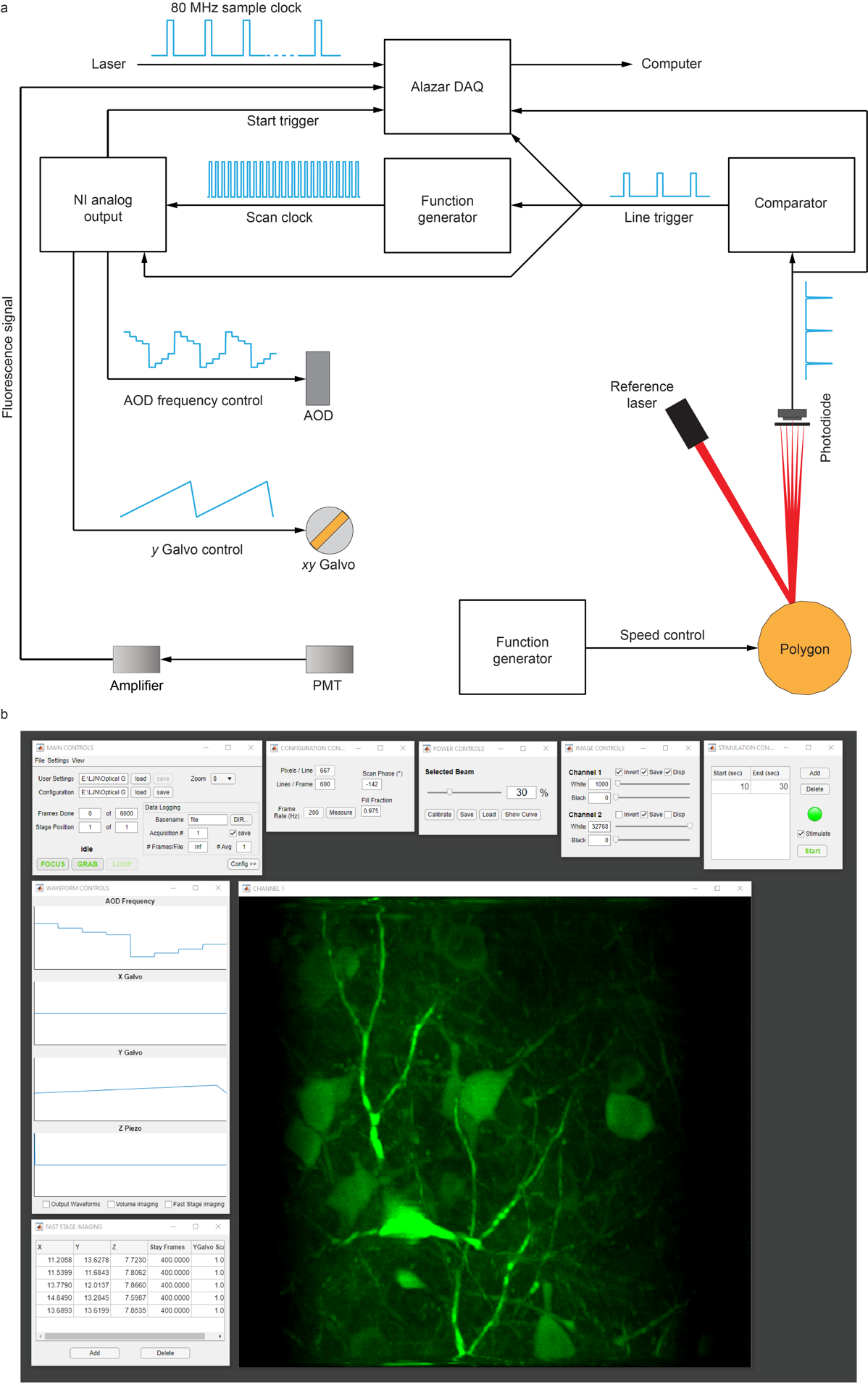


**Supplementary Figure 3 | Synchronization and data acquisition system design.** (a) Timing diagram of the data acquisitions system. AOD, acousto-optic deflector, DAQ, data acquisition; PMT, photomultiplier tube. (b) GUI of the data acquisition control program we wrote in MATLAB.


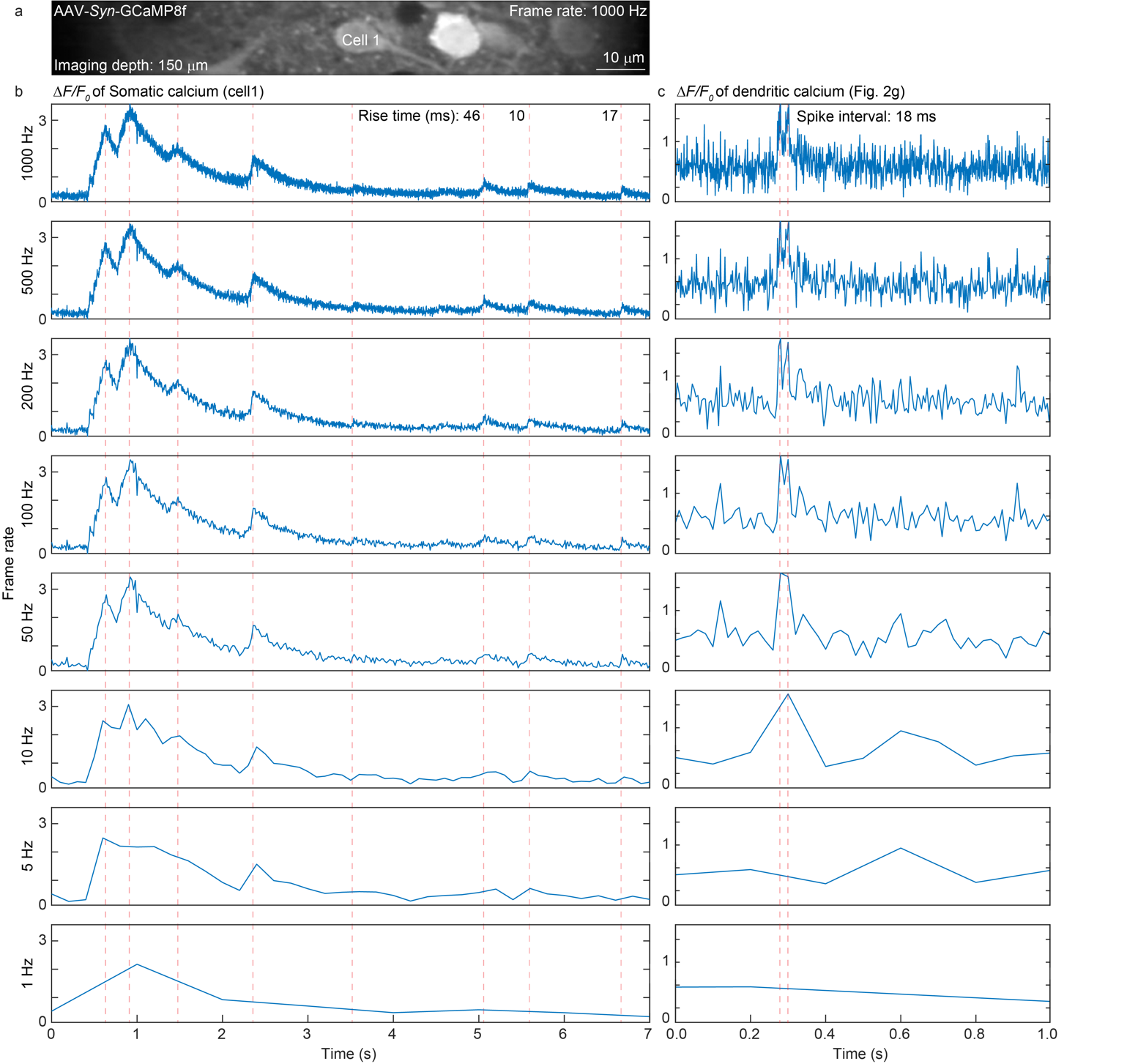


**Supplementary Figure 4 | Frame rate evaluation for imaging GCaMP8f expressing neurons.** (a) Calcium image of GCaMP8f expressing neurons. (b) Calcium transient of cell 1 at 1 kHz and at reduced rates by eliminating data points (e.g. 200 Hz is obtained by only keeping frame 1, 6, 11, ….). (c) Calcium transient of the dendritic structure in Fig. 2g at 1 kHz and at reduced rates by eliminating data points.


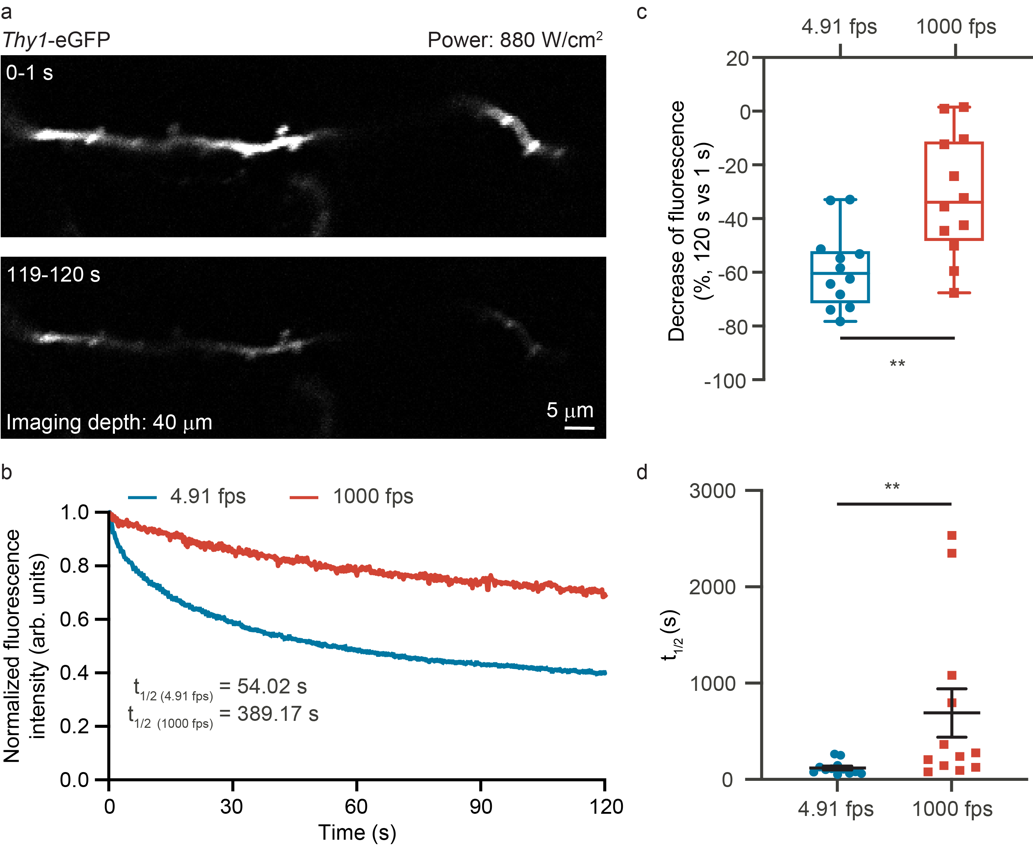


**Supplementary Figure 5 | Photobleaching comparison between the gearbox based high-rate imaging and the galvo based low-rate imaging with the same excitation parameters.** (a) Fluorescence images of dendrites in the brain of *Thy1*-eGFP mice. (b) Fluorescence signal decay over time recorded at different rates. t_1/2_, half time; fps, frames per second. (c) Statistical analysis of the fluorescence signal decay percentage over 120 seconds. For boxplots, minima and maxima are shown as the bounds of whiskers, and the centile, upper and lower quartiles are shown as the middle, top, and bottom lines of the box. n = 12 dendrites from 3 mice. ** *P* = 0.0021, two-sided unpaired t test. (d) Statistical analysis of fluorescence decay time. Data are represented as mean ± standard error of the mean. n = 12 dendrites from 3 mice. ** *P* = 0.0056, two-sided nonparametric Mann Whitney test.


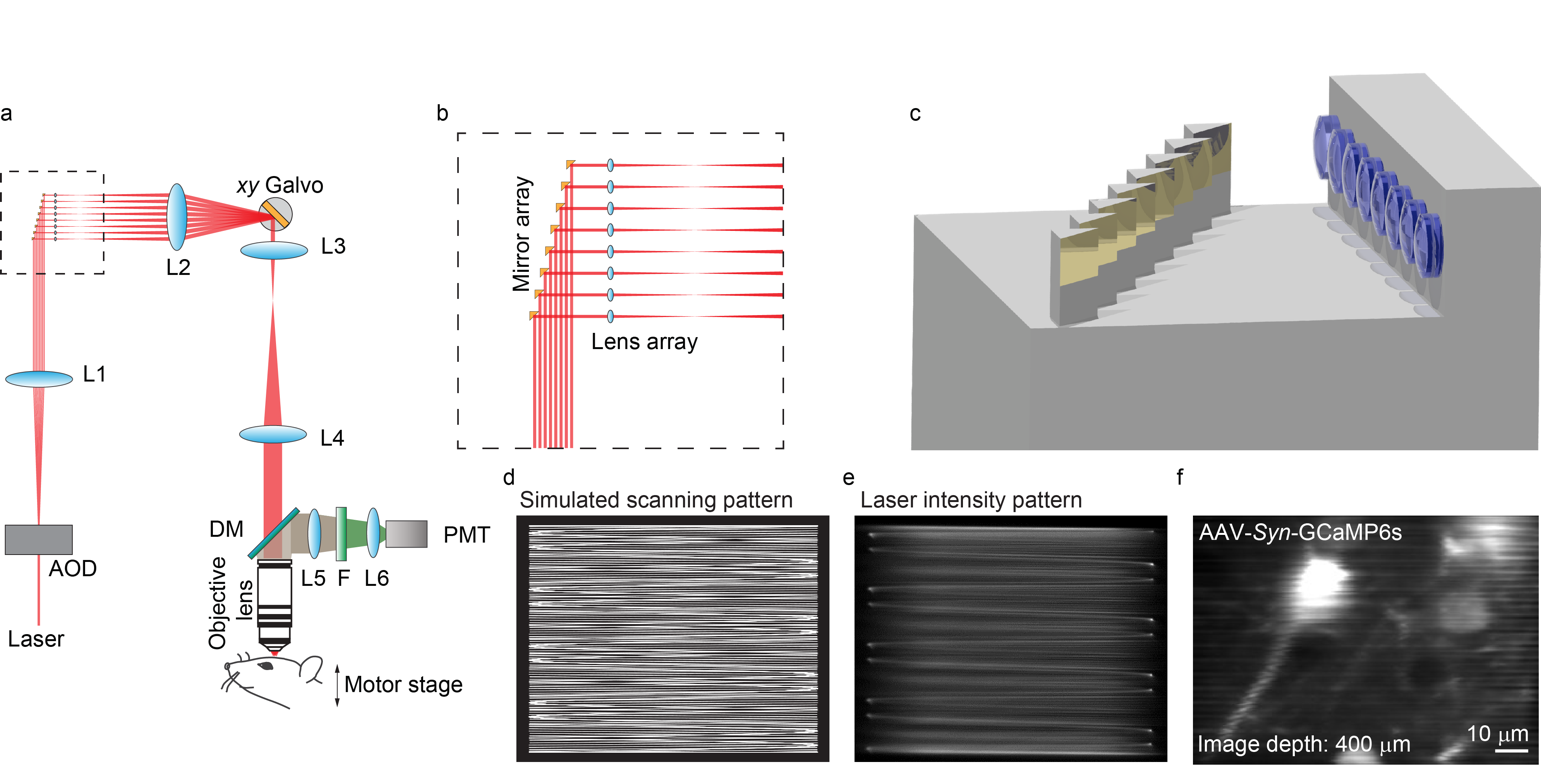


**Supplementary Figure 6 | Resonant galvo based optical gearbox system.** (a) System design of the resonant galvo based gearbox system. AOD, acousto-optic deflector; L1-6, optical lenses; DM, dichroic mirror; F, fluorescence bandpass filter; PMT, photomultiplier tube. (b) Zoomed-in view of the mirror array and lens array. (c) Mechanical holder for the mirror array and lens array. (d, e) Simulated and measured scanning pattern with the 8x gearbox setting, respectively. (f) Calcium imaging of neuronal structure at 200 Hz rate.

**Supplementary Table 1**

Summary of animals used in experiments

| Experiment | Age | Type | Sex |
| --- | --- | --- | --- |
| Fig. 2 | 4-6 weeks | 13 C57BL/6  (AAV-*Syn*-GCaMP8f,  AAV-*Syn*-cre,  AAV-*Syn-*FLEX-GCaMP8m) | 7 male, 6 females |
| Fig. 3 | 4-6 weeks | 4 C57BL/6 (AAV-*Syn*-GCaMP7f) | 2 male, 2 female |
| Fig. 4 | 4-6 weeks | 8 C57BL/6 | 4 male, 4 female |
| Supplementary Fig. 5 | 4-6 weeks | 3 *Thy1*-eGFP | 3 male |
| Supplementary Fig. 6 | 4-6 weeks | 9 C57BL/6 (AAV-*Syn*-GCaMP6s) | 5 male, 4 female |

**Supplementary Discussion 1**

The polygon scanner can support 55 k rpm with 18 facets and each facet is 10.7 mm long. For the dual-path configuration, each path just needs to be better than 50% in the duty cycle. So the maximum beam size can be 5.35 mm. Let’s assume we use 5.1 mm (a bit smaller than this physical limit). The angular resolvable spot at 920 nm is 0.92/5100 rad. With the two-photon excitation, the effective spot size is reduced by the square root of 2. So the spot size is 127.56 micro rad. Each 360-degree mechanical spin can yield a 720-degree optical scan. So the total number of resolvable spots per round is 98,516. With 55 k rpm, we have 90 million resolvable spots per second. To satisfy the sampling limit, we will need at least 180 million pixels per second.
