# Supplementary material for: Optical gearbox enabled versatile multiscale high-throughput multiphoton functional imaging
Source: Nat Commun. 2022 Nov 2;13:6564. doi: 10.1038/s41467-022-34472-6 (PMC9630539; doi:10.1038/s41467-022-34472-6)
Supplement: Supplementary file 2 — Description of Additional Supplementary Files [file 41467_2022_34472_MOESM2_ESM.pdf]

Description of Additional Supplementary Files

Supplementary Movie 1 | Polygon-based optical gearbox system with dual path configuration. The optical configuration and scanning sequence of the dual-path gearbox system.

Supplementary Movie 2 | In vivo 2D high-speed calcium recording with the gearbox system.

Supplementary Movie 3 | 3D volumetric recording with the gearbox system.

Supplementary Movie 4 | High-speed blood flow imaging with the gearbox system.
