# Supplementary material for: Optical gearbox enabled versatile multiscale high-throughput multiphoton functional imaging
Source: Nat Commun. 2022 Nov 2;13:6564. doi: 10.1038/s41467-022-34472-6 (PMC9630539; doi:10.1038/s41467-022-34472-6)
Supplement: Supplementary file 7 — Reporting Summary [file 41467_2022_34472_MOESM7_ESM.pdf]

nature portfolio

Corresponding author(s): Meng Cui

Last updated by author(s): Sep 19, 2022

Reporting Summary

Nature Portfolio wishes to improve the reproducibility of the work that we publish. This form provides structure for consistency and transparency in reporting. For further information on Nature Portfolio policies, see our Editorial Policies and the Editorial Policy Checklist.

Statistics

For all statistical analyses, confirm that the following items are present in the figure legend, table legend, main text, or Methods section.

n/a	Confirmed	
☐	☒	The exact sample size (n) for each experimental group/condition, given as a discrete number and unit of measurement
☐	☒	A statement on whether measurements were taken from distinct samples or whether the same sample was measured repeatedly
☐	☒	The statistical test(s) used AND whether they are one- or two-sided<br>*Only common tests should be described solely by name; describe more complex techniques in the Methods section.*
☒	☐	A description of all covariates tested
☒	☐	A description of any assumptions or corrections, such as tests of normality and adjustment for multiple comparisons
☐	☒	A full description of the statistical parameters including central tendency (e.g. means) or other basic estimates (e.g. regression coefficient) AND variation (e.g. standard deviation) or associated estimates of uncertainty (e.g. confidence intervals)
☐	☒	For null hypothesis testing, the test statistic (e.g. F, t, r) with confidence intervals, effect sizes, degrees of freedom and P value noted<br>*Give P values as exact values whenever suitable.*
☒	☐	For Bayesian analysis, information on the choice of priors and Markov chain Monte Carlo settings
☒	☐	For hierarchical and complex designs, identification of the appropriate level for tests and full reporting of outcomes
☒	☐	Estimates of effect sizes (e.g. Cohen's d, Pearson's r), indicating how they were calculated

Our web collection on statistics for biologists contains articles on many of the points above.

Software and code

Policy information about availability of computer code

Data collection	Custom software was used for data collection, which can be accessed in Science Data Bank [DOI: 10.57760/sciencedb.02764].
Data analysis	GraphPad Prism (8.0.2) and MATLAB 2021A were used for statistical analysis.

For manuscripts utilizing custom algorithms or software that are central to the research but not yet described in published literature, software must be made available to editors and reviewers. We strongly encourage code deposition in a community repository (e.g. GitHub). See the Nature Portfolio guidelines for submitting code & software for further information.

Data

Policy information about availability of data

All manuscripts must include a data availability statement. This statement should provide the following information, where applicable:

- Accession codes, unique identifiers, or web links for publicly available datasets
- A description of any restrictions on data availability
- For clinical datasets or third party data, please ensure that the statement adheres to our policy

The datasets generated and/or analyzed during the current study are available from the online repository Science Data Bank database [DOI: 10.57760/sciencedb.02730].

Human research participants

Policy information about studies involving human research participants and Sex and Gender in Research.

Reporting on sex and gender	not applicable
Population characteristics	not applicable
Recruitment	not applicable
Ethics oversight	not applicable

Note that full information on the approval of the study protocol must also be provided in the manuscript.

Field-specific reporting

Please select the one below that is the best fit for your research. If you are not sure, read the appropriate sections before making your selection.

[x] Life sciences [ ] Behavioural & social sciences [ ] Ecological, evolutionary & environmental sciences

For a reference copy of the document with all sections, see nature.com/documents/nr-reporting-summary-flat.pdf

Life sciences study design

All studies must disclose on these points even when the disclosure is negative.

Sample size	The imaging experiments were repeated on different mice so that the experiments can sufficiently demonstrate and characterize the imaging performance. All experiments were repeated on a minimum of 3 mice. No sample size calculation was performed as the imaging performance was similar on different animals. The samples used in the experiment have obtained consistent results, and the current sample size is sufficient to support the conclusions of this paper.
Data exclusions	No data exclusion.
Replication	Each type of animal experiment was repeated on at least 3 mice, which showed consistent results.
Randomization	Both male and female mice were used in the imaging experiment. There was no difference in the imaging performance. Randomization is not relevant to this study as the imaging performance is independent of the animal. There were no control group and experiment group distinctions in this work.
Blinding	No blinding was used as the imaging performance was independent of animals. There were no control group and experiment group distinctions in this work.

Reporting for specific materials, systems and methods

We require information from authors about some types of materials, experimental systems and methods used in many studies. Here, indicate whether each material, system or method listed is relevant to your study. If you are not sure if a list item applies to your research, read the appropriate section before selecting a response.

Materials & experimental systems

n/a	Involved in the study	
x		Antibodies
x		Eukaryotic cell lines
x		Palaeontology and archaeology
	x	Animals and other organisms
x		Clinical data
x		Dual use research of concern

Methods

n/a	Involved in the study	
x		ChIP-seq
x		Flow cytometry
x		MRI-based neuroimaging

Animals and other research organisms

Policy information about studies involving animals; ARRIVE guidelines recommended for reporting animal research, and Sex and Gender in Research

Laboratory animals	The wild-type (WT) C57BL/6 mice (for virus injection), Thy1-eGFP M line were purchased from the Jackson Laboratory. The wild-type (WT) C57BL/6 mice (for GCaMP virus injection), transgenic Thy1-eGFP mice were used in this study. The surgical procedures were performed on one to two months old male and female mice. The housing for mice had a 12 hour on/off light cycle. Lights were on at 6am and off at 6pm. Temperatures for mice were kept between 20 and 25 degrees Celsius. Humidity was kept in the range of 30-70 percent.
Wild animals	No wild animals were used in the study.
Reporting on sex	Both male and female mice were used in the imaging experiment. The results were independent of animals.
Field-collected samples	No field collected samples were used in the study.
Ethics oversight	All procedures involving mice were approved by the Animal Care and Use Committees of Purdue University.

Note that full information on the approval of the study protocol must also be provided in the manuscript.
